# Tuning the singlet-triplet energy gap: a unique approach to efficient photosensitizers with aggregation-induced emission (AIE) characteristics†
†Electronic supplementary information (ESI) available: Synthesis and characterization of the intermediates and molecular orbital data. See DOI: 10.1039/c5sc01733e



**DOI:** 10.1039/c5sc01733e

**Published:** 2015-07-17

**Authors:** Shidang Xu, Youyong Yuan, Xiaolei Cai, Chong-Jing Zhang, Fang Hu, Jing Liang, Guanxin Zhang, Deqing Zhang, Bin Liu

**Affiliations:** a Department of Chemical and Biomolecular Engineering , National University of Singapore , 4 Engineering Drive 4 , Singapore 117585; b Beijing National Laboratory for Molecular Sciences , Organic Solids Laboratory , Institute of Chemistry , Chinese Academy of Sciences , Beijing 100190 , China; c Institute of Materials Research and Engineering , 3 Research Link , Singapore 117602 . Email: cheliub@nus.edu.sg

## Abstract

The efficiency of the intersystem crossing process can be improved by reducing the energy gap between the singlet and triplet excited states (Δ*E*_ST_), which offers the opportunity to improve the yield of the triplet excited state. Herein, we demonstrate that modulation of the excited states is also an effective strategy to regulate the singlet oxygen generation of photosensitizers. Based on our previous studies that photosensitizers with aggregation-induced emission characteristics (AIE) showed enhanced fluorescence and efficient singlet oxygen production in the aggregated state, a series of AIE fluorogens such as **TPDC**, **TPPDC** and **PPDC** were synthesized, which showed Δ*E*_ST_ values of 0.48, 0.35 and 0.27 eV, respectively. A detailed study revealed that **PPDC** exhibited the highest singlet oxygen efficiency (0.89) as nanoaggregates, while **TPDC** exhibited the lowest efficiency (0.28), inversely correlated with their Δ*E*_ST_ values. Due to their similar optical properties, **TPDC** and **PPDC** were further encapsulated into nanoparticles (NPs). Subsequent surface modification with cell penetrating peptide (TAT) yielded **TAT–TPDC NPs** and **TAT–PPDC NPs**. As a result of the stronger singlet oxygen generation, **TAT–PPDC NPs** showed enhanced cancer cell ablation as compared to **TAT–TPDC NPs**. Fine-tuning of the singlet-triplet energy gap is thus proven to be an effective new strategy to generate efficient photosensitizers for photodynamic therapy.

## Abstract 2

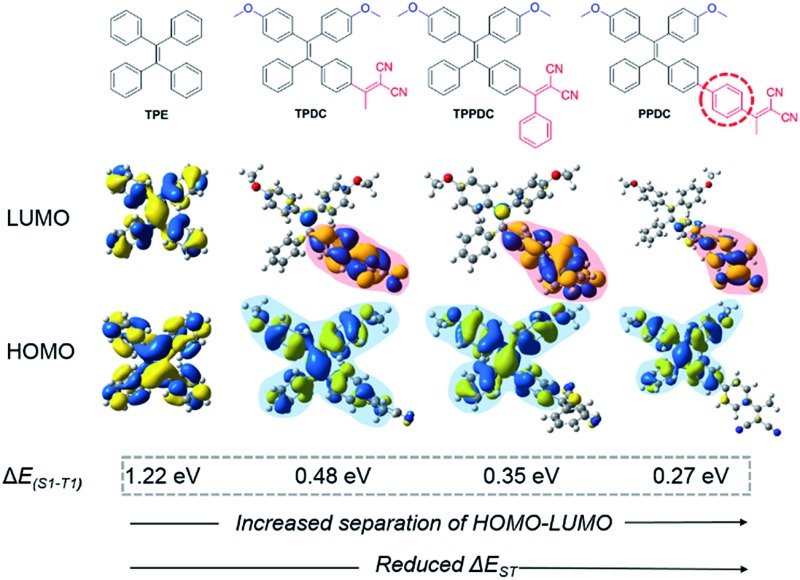
The efficiency of the intersystem crossing process can be improved by reducing the energy gap between the singlet and triplet excited states (Δ*E*_ST_), which offers the opportunity to improve the yield of the triplet excited state.

## Introduction

Photosensitized generation of reactive oxygen species (ROS) has attracted great research interest, and has found numerous applications in different fields,^1,2^ including synthetic organic chemistry,^3^ wastewater treatment,^5,6^ and photodynamic therapy (PDT).^4^ PDT represents a well-consolidated but gradually expanding approach to the treatment of cancer. It involves excitation of a photosensitizer (PS) with specific light wavelengths, which is followed by intersystem crossing (ISC) from its lowest singlet excited state (S_1_) to the lowest triplet excited state (T_1_); subsequently, energy transfer from the T_1_ of the PS to ground-state oxygen (^3^O_2_) generates ROS, which causes oxidative damage of targets.^7^

The primary cytotoxic agent involved in this photodynamic process is singlet oxygen (^1^O_2_), the efficient generation of which is habitually related to the ISC efficiency of the sensitizer and concentration quenching of the excited state. As such, efficient ISC offers the opportunity to improve the triplet excited state yield, which is highly desirable for efficient ^1^O_2_ generation. In addition, any system with minimized concentration quenching of the excited state is also favorable.

**Scheme 1:**
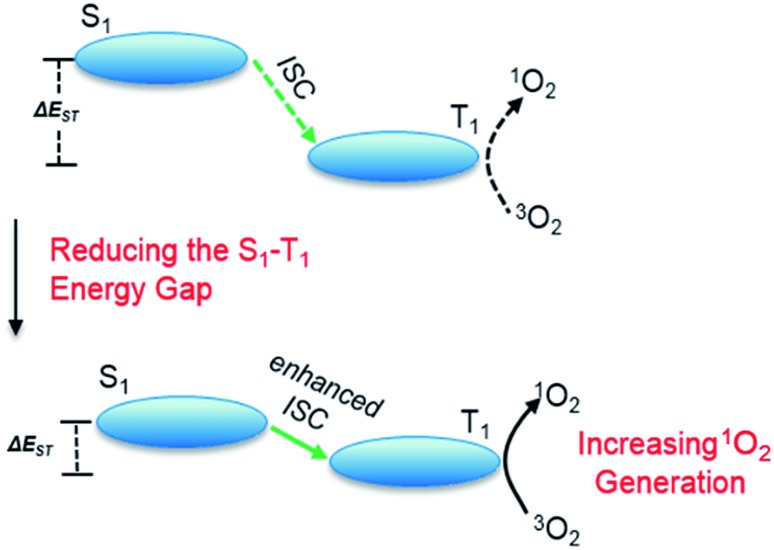
A physical model of ^1^O_2_ generation, depicting the S_1_–T_1_ ISC process and the proposed strategy for increasing ^1^O_2_ generation.

Incorporation of heavy atoms into molecular structures to enhance the spin–orbit perturbations is one of the most widely used approaches to improve the ISC efficiency of PSs.^8–10^ However, the presence of heavy atoms such as selenium, iodine, bromine, and certain lanthanides has often been reported to cause increased “dark toxicity”.^11^ It is thus important to propose alternative strategies to achieve strong ISC without using heavy atoms, to minimize dark toxicity. Previous studies have shown that the ISC rate constants could be estimated from eqn (1),^12^ where *H*_SO_ is the Hamiltonian for the spin–orbit perturbations (SOP) and Δ*E*_S_1_–T_1__ (Δ*E*_ST_) is the energy gap between the S_1_ and T_1_ states. ISC can be modeled by mixing of T_1_ with S_1_ states due to SOP. This equation shows the relationship between ISC rate and Δ*E*_ST_, which paves the way for improving ISC efficiency, and consequently ^1^O_2_ generation (Scheme 1).1
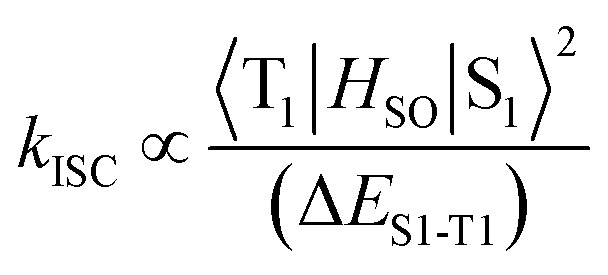



Concentration quenching of the excited state is another common problem that affects the ^1^O_2_ generation efficiency of PSs. This is particularly serious for hydrophobic PSs, which tend to aggregate in aqueous media due to their rigid planar structures, resulting in aggregation-caused quenching (ACQ)^13^ and remarkable reduction in ^1^O_2_ generation efficiency.^14^ The quenching is more severe when the PSs, such as porphyrin derivatives, are encapsulated into nanocarriers, which could lead to significantly decreased fluorescence and photodynamic efficiency.^15^ Opposite to the ACQ effect, propeller-shaped fluorogens with aggregation-induced emission (AIEgen) characteristics have recently emerged as a powerful and versatile tool for biomedical applications.^16–25^ AIEgens are almost non-emissive in the molecular state but can be induced to emit strong fluorescence in the aggregated state owing to the prohibition of energy dissipation through non-radiative channels.^26–33^ In addition, AIEgen PSs have been found to exhibit enhanced fluorescence and efficient photosensitizing characteristics in the solid state,^24,34–38^ which paves the way for tackling the ACQ problem of PSs. This motivates us to develop more efficient AIEgen PSs for image-guided photodynamic therapy.

To develop more efficient AIEgen PSs, we propose to control the Δ*E*_ST_ values by incorporation of electron donors and acceptors into π-conjugated systems to manipulate the HOMO–LUMO distribution.^39–42^ By taking advantage of the unique optical properties of AIEgens, in this contribution, a series of AIEgens with methoxy as the electron donor and dicyanovinyl as the acceptor were synthesized, and their ability to generate ^1^O_2_ was studied both in solution and in cells. The work demonstrates for the first time that reducing Δ*E*_ST_ is an effective strategy for achieving efficient PSs for photodynamic therapy.

## Experimental section

### Materials

1,2-Distearoyl-*sn-glycero*-3-phosphoethanolamine-*N*-maleimide(polyethyleneglycol)-3000] (DSPE-PEG_3000_-Mal) was ordered from Avanti Polar Lipids. 4-Acetylphenylboronic acid, malononitrile, 2′,7′-dichlorodihydrofluorescein diacetate (DCF-DA) and other chemicals were all purchased from Alfa Aesar or Sigma-Aldrich. Cell penetrating peptide HIV-1 TAT was purchased from GL Biochem Ltd. Trypsin–EDTA, Annexin V-FITC, fetal bovine serum (FBS) and Hoechst 33342 were purchased from Life Technologies. **TPDC** and **TPPDC** were synthesized according to our previous reports.^35,37^

### Instrumentation

NMR spectra were performed on a Bruker ARX 400 NMR spectrometer. Electrospray ionization mass spectrometry (ESI-MS) was performed on a Proteome X-LTQ. Particle size and size distribution were determined by laser light scattering (LLS) with a particle size analyzer (90 Plus, Brookhaven Instruments Co., United States) at a fixed angle of 90° at room temperature. The zeta potential was determined by a Malvern Zetasizer Nano ZS90 (Worcestershire, UK). TEM images were obtained from a JEOL JEM-2010 transmission electron microscope with an accelerating voltage of 200 KV. UV-vis absorption spectra were taken on a Shimadzu Model UV-1700 spectrometer. Photoluminescence (PL) spectra were measured on a Perkin-Elmer LS 55 spectrofluorometer. All UV and PL spectra were collected at 24 ± 1 °C.

### Computational details

All molecules were fully optimized by the hybrid B3LYP, in combination with the 6-31G(d) basis set. The excited-state characteristics were calculated by time-dependent density functional theory (TD-DFT) using optimized ground state geometries. TD-DFT in combination with the B3LYP hybrid functional method and the 6-31G(d) basis set has been shown to provide accurate energies for the excited state of D–A molecular systems with less than 0.1 eV error.^39–41^ The geometry data of **TPDC**, **TPPDC** and **PPDC** are provided in the (ESI).†

### Synthesis of **PPDC**

The synthesis and characterization of the intermediates of **DMTPBr** and **PPAc** are described in the ESI.† To synthesize **PPDC**, **PPAc** (100 mg, 0.18 mmol), malononitrile (100 mg, 1.50 mmol) and ammonium acetate (139 mg, 1.80 mmol) were dissolved in a mixture of dichloromethane (20 mL) and methanol (4 mL). Then silica gel (2.4 g) was added to the above mixture, and the solvent was removed under reduced pressure. The resulting mixture was heated at 100 °C for 4 h. The mixture was cooled down and subsequently separated by chromatography (hexane/ethyl acetate = 20/1, v/v) to give the desired product as a yellow powder (74 mg, 73.6% yield). ^1^H NMR (400 MHz, CDCl_3_) *δ* 7.65–7.50 (m, 4H), 7.31 (d, *J* = 8.4 Hz, 2H), 7.11–6.95 (m, 7H), 6.92–6.80 (m, 4H), 6.65–6.49 (m, 4H), 3.67 (d, *J* = 0.7 Hz, 6H), 2.59 (s, 3H). ^13^C NMR (100 MHz, CDCl_3_) *δ* 174.5, 158.30, 158.2, 144.8, 144.8, 144.0, 140.9, 138.4, 136.4, 136.1, 134.2, 132.6, 132.5, 132.0, 131.4, 128.0, 127.8, 127.2, 126.3, 113.1, 113.0, 112.9, 83.8, 77.3, 77.0, 76.7, 60.3, 55.0, 55.0, 23.9, 21.0, 14.1; EI-MS, *m*/*z*: [M + 1]^+^ calcd 558.2, found 558.5.

### Preparation of **TAT–TPDC NPs** and **TAT–PPDC NPs**

DSPE-PEG_3000_-Mal (1.0 mg) and **TPDC** (or **PPDC**) (0.5 mg) in THF solution (1 mL) was poured into water (10 mL) and sonicated for 2 min with a microtip probe sonicator at 12 W output. The organic solvent was removed by stirring the mixture at room temperature to yield NPs. The NPs were further reacted with HIV-TAT peptide (RKKRRQRRRC, 1.5 mg) *via* click coupling reaction overnight. Then the TAT-functionalized NPs were purified by ultrafiltration (MWCO 20 000) three times, and resuspended in Milli-Q water for further use. The grafting efficiency of TAT to the NPs was evaluated using the following procedure. In brief, the filtrate containing the unreacted TAT was carefully collected and analyzed using HPLC. The amount of grafted TAT was obtained by subtracting the amount of TAT in the filtrate from the total amount of TAT used. The grafting efficiency of TAT to the NPs was calculated by taking the ratio between the amount of grafted TAT and the initial amount of TAT used for conjugation.

### ^1^O_2_ quantum yield measurements^44^

The ^1^O_2_-sensitive indicator 9,10-anthracenediyl-bis(methylene) dimalonic acid (ABDA) was used as the ^1^O_2_ indicator, and Rose Bengal (RB) was employed as the standard photosensitizer. In these experiments, 10 μL of ABDA solution (2 M) was added to 1 mL of sample solution, and white light (400–800 nm) with a power density of 0.25 W cm^–2^ was used as the irradiation source. The absorbance of ABDA at 378 nm was recorded at different irradiation times to obtain the decay rate of the photosensitizing process. The ^1^O_2_ quantum yield of the PS in water (*Ф*_PS_) was calculated using the following formula:2
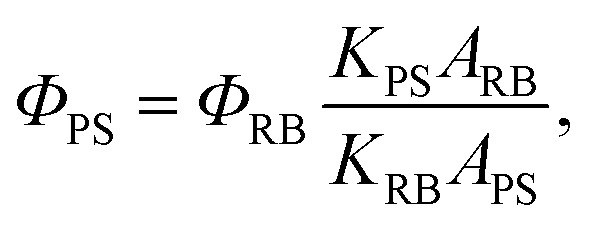
where *K*_PS_ and *K*_RB_ are the decomposition rate constants of ABDA by the PSs and RB, respectively. *A*_PS_ and *A*_RB_ represent the light absorbed by the PSs and RB, respectively, which are determined by integration of the areas under the absorption bands in the wavelength range of 400–800 nm. *Ф*_RB_ is the ^1^O_2_ quantum yield of RB, which is 0.75 in water.

### Cell culture

Human cervix carcinoma HeLa cells were provided by American Type Culture Collection (ATCC) and cultured in DMEM medium containing penicillin (100 U mL^–1^), FBS (Invitrogen, 10%) and streptomycin (100 μg mL^–1^). The cells were maintained in a humidified incubator with 5% CO_2_ at 37 °C.

### Quantification of the cellular uptake by fluorescence microplate reader

HeLa cells in 96-well plates were incubated with the medium containing **TAT–TPDC NPs** and **TAT–PPDC NPs** (5 μg mL^–1^) for a designated time. Then the cells were washed with 1 × PBS twice and the fluorescence intensity was studied using a T-CAN microplate reader with excitation and emission wavelengths at 405 and 595 nm, respectively.

### Confocal imaging

The cells were cultured in 8-well chambers at 37 °C and precultured overnight. Then the culture medium was removed and the cells were washed with 1 × PBS before incubation with the **TAT–TPDC NPs** and **TAT–PPDC NPs** (5 μg mL^–1^). The cell nuclei were living stained with Hoechst 33342 (Life Technologies, *E*_x_: 404 nm, *E*_m_: 430–470 nm). The cells were imaged by confocal laser scanning microscope (CLSM, Zeiss LSM 410, Jena, Germany). The images were analyzed with the Image J 1.43 × program (developed by the NIH, ; http://rsbweb.nih.gov/ij/).

### Cytotoxicity studies

The metabolic activity of the cells was assessed by 3-(4,5-dimethythiazol-2-yl)-2,5-diphenyltetrazolium bromide (MTT) assays. After 24 h incubation in DMEM medium, the cells were incubated with different concentrations of **TAT–TPDC NPs** and **TAT–PPDC NPs** for 4 h, then the medium was replaced with a fresh one and exposed to white light irradiation. The cells were further incubated for 24 h and washed with 1 × PBS before the addition of 100 μL of MTT solution (0.5 mg mL^–1^) into each well. After 3 h incubation, the MTT solution was removed and DMSO (100 μL) was added into each well. The absorbance of MTT at 570 nm was studied with the microplate reader (Genios Tecan). Cells without any treatment were used as a control.

## Results and discussion

### Molecular design and TD-DFT calculation of Δ*E*_ST_

**Fig. 1:**
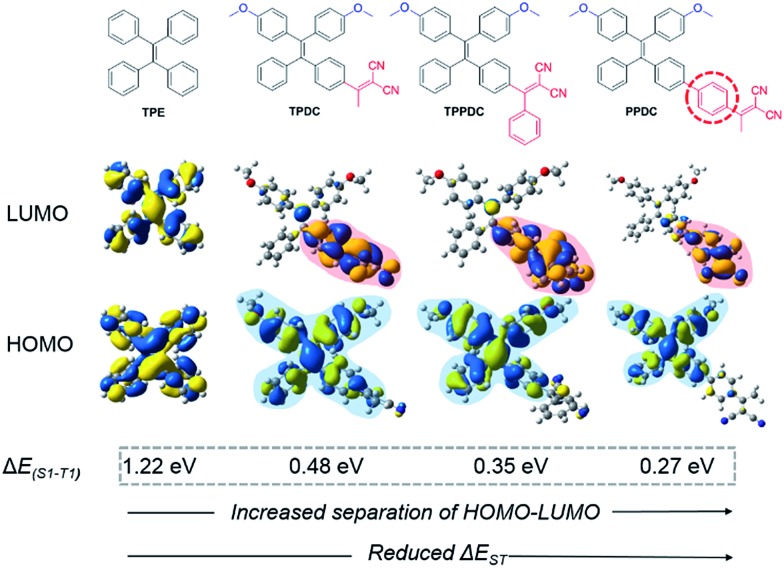
Chemical structures and HOMO–LUMO distributions of **TPE**, **TPDC**, **TPPDC** and **PPDC**, optimized structures of the HOMO and LUMO at S_1_ were calculated by TD-DFT (Gaussian 09/B3LYP/6-31G(d)).

The molecular designs of **TPDC**, **TPPDC** and **PPDC** are based on the following considerations: (1) tetraphenylethylene (**TPE**) is AIE-active, and the AIE characteristics can be retained after chemical modification;^27,29^ (2) small Δ*E*_ST_ values can be realized *via* intramolecular charge transfer within molecular systems containing spatially separated donor and acceptor moieties;^39–42^ (3) the benzene ring is a widely used π bridge for HOMO–LUMO engineering; (4) close molecular structures suggest a similar amount of spin–orbit coupling between S_1_ and T_1_, so that Δ*E*_ST_ can be viewed as inversely proportional to *k*_ISC_ (eqn (1)). Accordingly, based on the parent **TPE**, by incorporating dicyanovinyl as the electron acceptor and methoxy as the electron donor, **TPDC**, **TPPDC** and **PPDC** were synthesized and purified with high yields. The molecular structures, HOMO and LUMO distribution and Δ*E*_ST_ values of all three compounds are summarized in Fig. 1. As predicted by time-dependent DFT (TD-DFT), the Δ*E*_ST_ values of **TPDC**, **TPPDC** and **PPDC** are 0.48, 0.35 and 0.27 eV, respectively, which are much smaller than that for **TPE** (1.22 eV). The small Δ*E*_ST_ suggests that a potentially high ISC rate is possible for efficient ^1^O_2_ generation.

### Synthesis

**Scheme 2:**
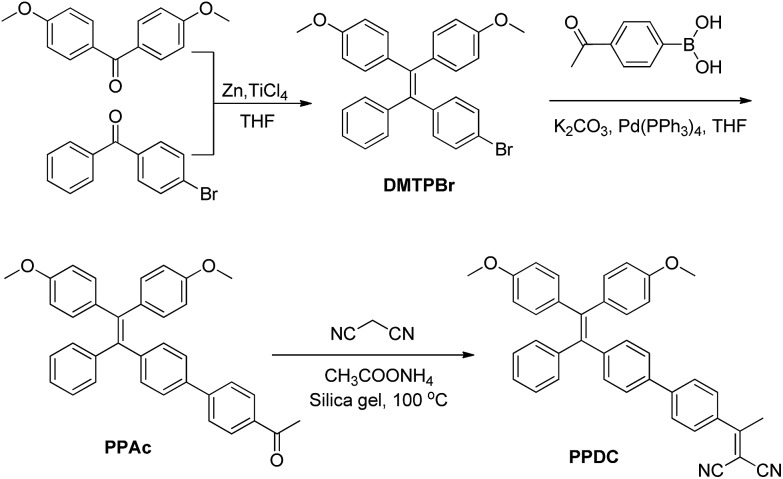
Synthetic route to **PPDC**.

The synthesis of **PPDC** is shown in Scheme 2. **DMTPBr** was synthesized according to the reported procedures.^35^ **DMTPBr** was first transformed into **PPAc** *via* Suzuki-coupling with 4-acetylphenylboronic acid. **PPDC** was subsequently obtained in 74.6% yield by the reaction between **PPAc** and malononitrile in the presence of ammonium acetate and pyridine. The final product and intermediates were characterized by NMR and mass spectrometry in Fig. S1–S8,† which revealed their correct structures with high purity.

### Optical properties and ^1^O_2_ generation of photosensitizers

**Fig. 2:**
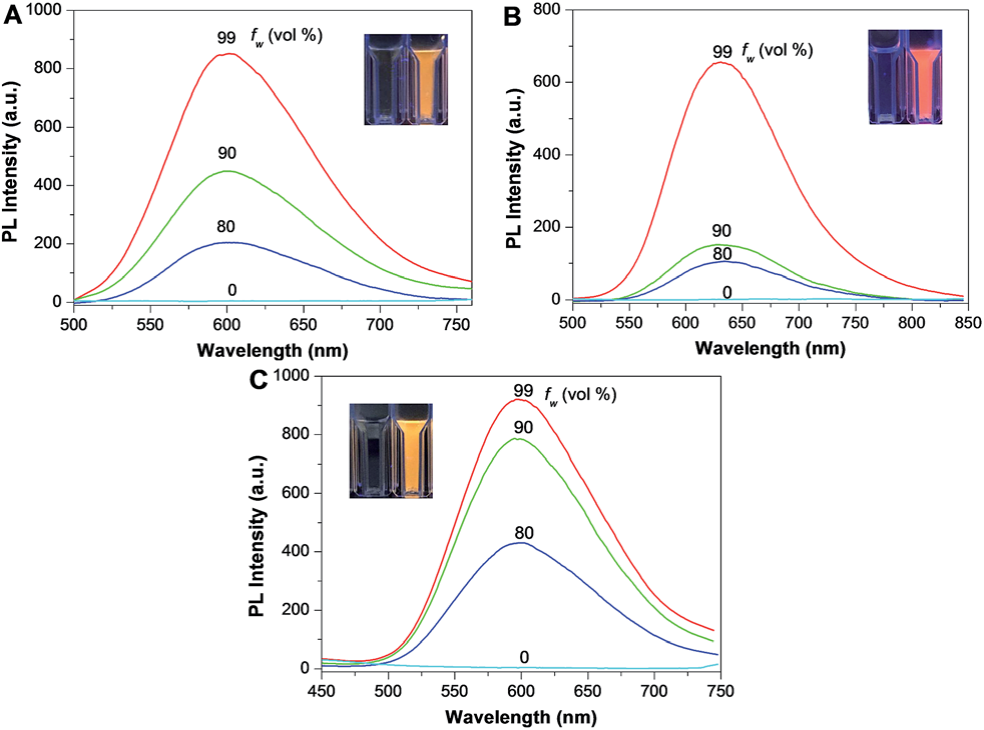
Fluorescence spectra of **TPDC** (A), **TPPDC** (B), **PPDC** (C) in DMSO/water mixtures with different volume fractions of water at a concentration of 10 μM. The excitation wavelengths for (A), (B) and (C) are 400, 390, 420 nm, respectively. The insets show photographs of the compounds in DMSO and DMSO : water (v : v, 1 : 99) under UV light (365 nm) illumination.

**Fig. 3:**
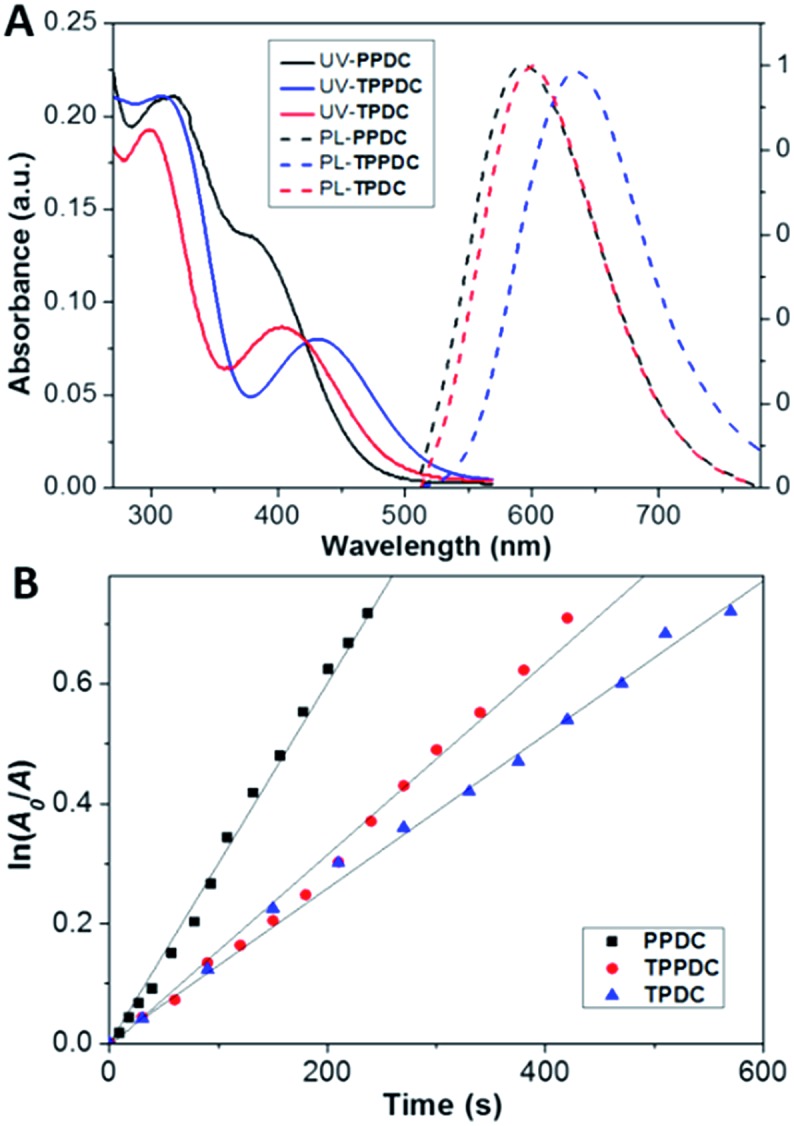
(A) UV-vis absorption (solid lines) and normalized photoluminescence (PL) spectra (dashed lines) of 10 μM each of **PPDC**, **TPDC** and **TPPDC** in DMSO/water (v/v = 1/99). (B) The decomposition rates of ABDA by **PPDC**, **TPDC** and **TPPDC**; *A*_0_ and *A* are the absorbance of ABDA in the presence of the AIEgen PSs at 378 nm before and after irradiation, respectively.

The optical properties of **TPDC**, **TPDCP** and **PPDC** were investigated first. The AIE characteristics of all the compounds were studied by monitoring their emission properties in DMSO/water mixtures with different volume fractions of water. As shown in Fig. 2, all three compounds are almost non-emissive in pure DMSO and they gradually become emissive after introducing a certain amount of water into the DMSO solution, indicating that they are indeed AIE active. The size distributions of the aggregates formed in DMSO/water (v/v = 1/99) mixtures were evaluated by laser light scattering (LLS), which revealed an average size of ∼50 nm for each (Fig. S9†). Fig. 3A shows the absorption and emission spectra of **TPDC**, **TPDCP** and **PPDC** as nanoaggregates at the same concentration of 10 μM. Although **TPDC** shows a red-shifted absorption spectrum as compared to **PPDC**, they have similar light absorption in the white light range (>400 nm) by comparing the areas under the absorption spectra.

To assess the capabilities of **PPDC**, **TPDC** and **TPPDC** for ^1^O_2_ generation, a commercial ^1^O_2_ probe, ABDA, was used as an indicator, and RB was used as the standard PS (the ^1^O_2_ quantum yield for *Ф*_RB_ is 0.75 in water).^43^ As shown in Fig. S10,† under white light irradiation, the presence of **PPDC**, **TPDC**, **TPPDC** or RB, in the ABDA solution leads to gradually decreased ABDA absorbance with prolonged irradiation time, indicating degradation of ABDA by the generated ^1^O_2_ in solution. It is important to note that under the same conditions, the white light irradiation on ABDA alone does not lead to an obvious change in absorbance. If we define *A*_0_ and *A* as the absorbance of ABDA in the presence of these AIEgen PSs at 378 nm before and after irradiation, respectively, the plot of ln (*A*_0_/*A*) against time gives straight lines in Fig. 3B. From the slopes, the decomposition rate constants (*K*_PS_) of **PPDC**, **TPPDC** and **TPDC** can be calculated as 0.0032, 0.0018 and 0.0013 s^–1^, respectively. Under the same experimental conditions, the rate constant for RB (*K*_RB_) is 0.0055 s^–1^ (Fig. S10†). The integrations of the optical absorption in the wavelength range of 400–800 nm (*A*_PS_) for RB, **PPDC**, **TPPDC** and **TPDC** are 9.52, 4.68, 7.33 and 5.67, respectively (Fig. S10†). Interestingly, **PPDC** shows the second largest *K*_PS_ with the smallest integrated area. The ^1^O_2_ quantum yields of **PPDC**, **TPPDC** and **TPDC** were calculated to be 0.89, 0.32 and 0.28, respectively. These results agreed well with the prediction based on eqn (1). Tuning the Δ*E*_ST_ is thus an effective strategy to yield PSs with controlled ^1^O_2_ generation efficiency. Due to the similar *A*_PS_ values for **TPDC** and **PPDC**, they were chosen for ^1^O_2_ generation comparison in cells.

### Preparation and characterization of **TAT–TPDC NPs** and **TAT–PPDC NPs**

**Scheme 3:**
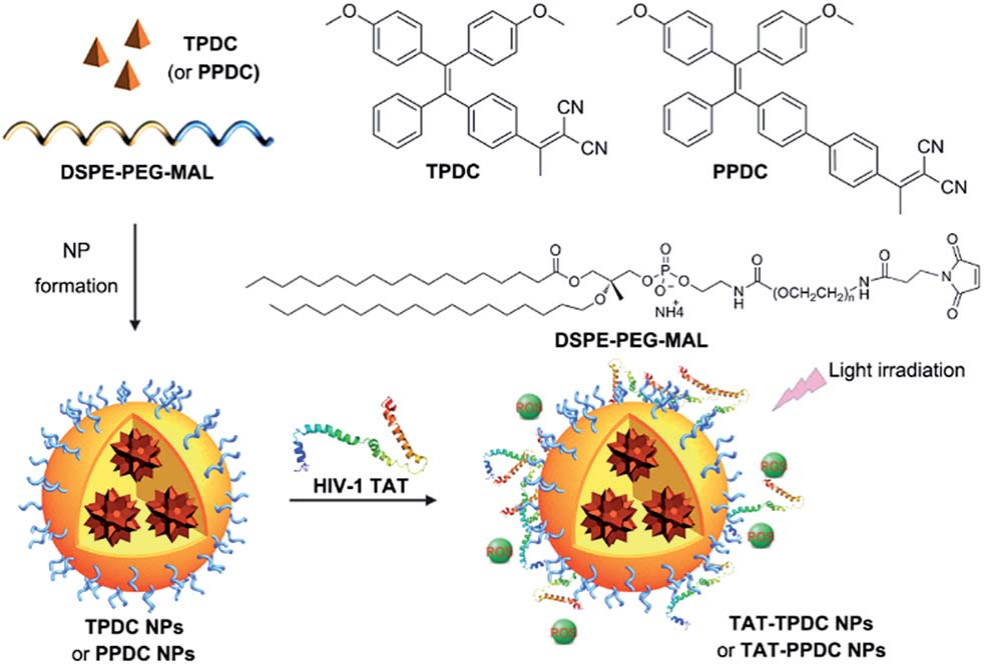
Schematic illustration of AIE NP formation and surface modification with TAT.

The **TAT–TPDC NPs** and **TAT–PPDC NPs** were prepared *via* the modified nano-precipitation method (Scheme 3).^19^ The maleimide-bearing copolymer DSPE-PEG_3000_-Mal was used as the matrix to encapsulate **TPDC** and **PPDC** to yield maleimide-functionalized AIE NPs, which were subsequently reacted with sulfhydryl (–SH) bearing cell-penetrating peptide (RKKRRQRRRC) to yield the **TAT–TPDC NPs** and **TAT–PPDC NPs**, respectively.

**Fig. 4:**
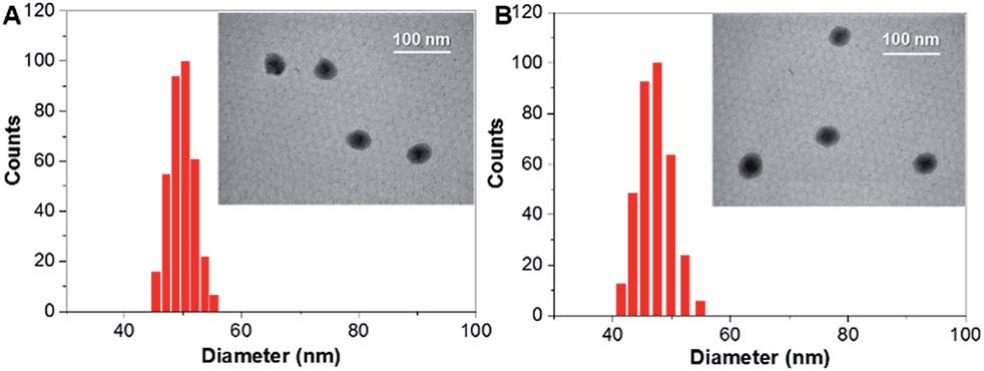
Size distribution and TEM images (inset) of **TAT–TPDC NPs** (A) and **TAT–PPDC NPs** (B).

The absorption and emission spectra of **TAT–TPDC NPs** and **TAT–PPDC NPs** are shown in Fig. S11.† When the same concentrations of TAT and NPs were used, the TAT conjugation efficiencies were calculated to be 62 ± 5% and 58 ± 4% for **TAT–TPDC NPs** and **TAT–PPDC NPs**, respectively. The size distributions of both NPs were evaluated by laser light scattering (LLS), which revealed an average size of ∼50 nm for each (Fig. 4). Likewise, **TAT–TPDC NPs** and **TAT–PPDC NPs** exhibit similar morphology according to the TEM images shown in the insets of Fig. 4. In addition, the zeta potentials for **TAT–TPDC NPs** and **TAT–PPDC NPs** are 23.7 mV and 23.4 mV, respectively. In view of the similar TAT conjugation efficiency, particle size, and surface charge of **TAT–TPDC NPs** and **TAT–PPDC NPs**, it is reasonable to expect that the two NPs would show similar uptake efficiency by the same cancer cells.

### Imaging of live cancer cells

**Fig. 5:**
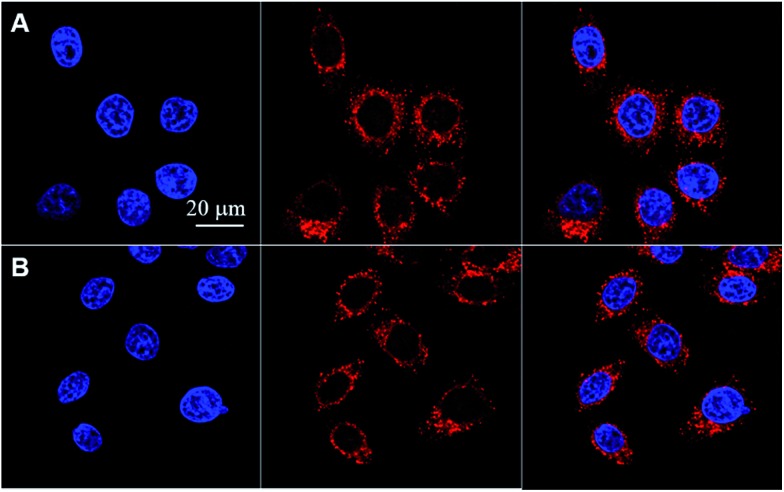
Confocal images of HeLa cells upon incubation with **TAT–TPDC NPs** (A) and **TAT–PPDC NPs** (B) for 4 h. The red fluorescence is from NPs and the blue fluorescence is from Hoechst 33342-stained cell nuclei. All the images share the same scale bar of 20 μm.

**TAT–TPDC NPs** and **TAT–PPDC NPs** were incubated with human cervix carcinoma HeLa cells for 4 h and their confocal fluorescence images are shown in Fig. 5. Red fluorescence either from **TAT–TPDC NPs** or **TAT–PPDC NPs** in the cell cytoplasm and blue fluorescence from Hoechst in the cell nuclei are simultaneously observed. Further quantitative fluorescence intensity analysis of both samples of NPs was performed by comparing the fluorescence intensity ratio (**TPDC**/**PPDC**) of the NPs retained in the medium before and after cell incubation and cell removal. The same intensity ratio indicates that the HeLa cell uptake of both types of NPs is similar.

### Photodynamic therapy

**Fig. 6:**
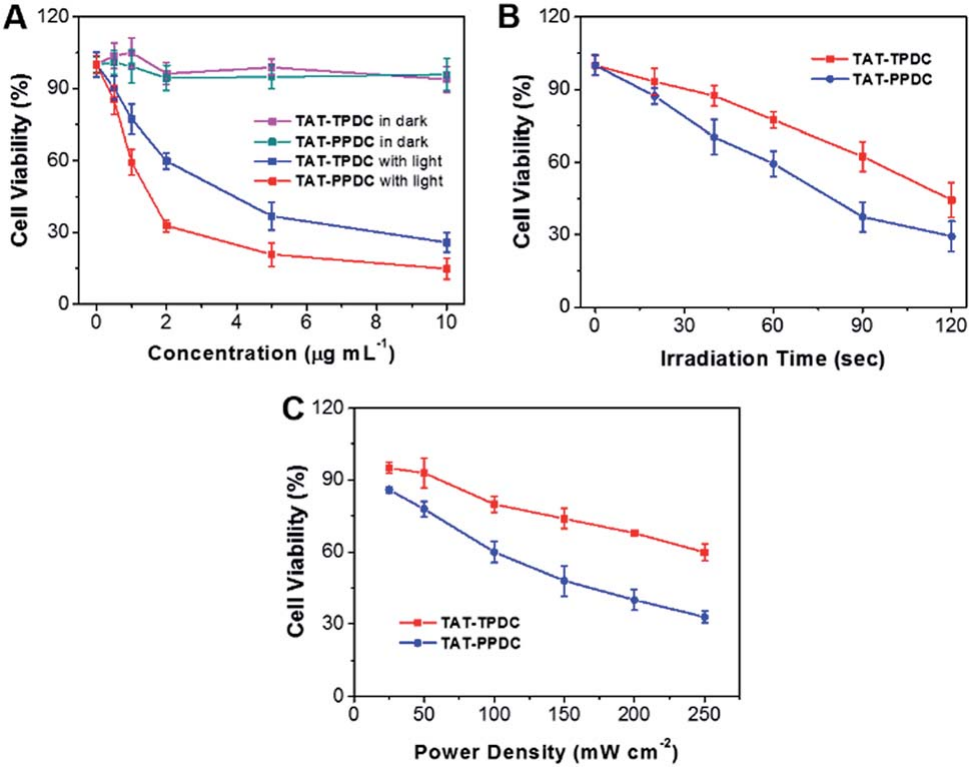
(A) The viability of HeLa cells upon treatment with **TAT–TPDC NPs** and **TAT–PPDC NPs** with or without light irradiation and further incubation for 24 h in fresh medium. The viability of HeLa cells incubated with **TAT–TPDC NPs** and **TAT–PPDC NPs** for different durations of light irradiation (B) or at different power densities (C), followed by further incubation for 24 h in fresh medium. Data represent mean values ± standard deviation, *n* = 3.

Low cytotoxicity in dark conditions but high toxicity upon exposure to light irradiation is essential for phototherapy. Quantitative evaluation of the therapeutic effect of **TAT–TPDC NPs** and **TAT–PPDC NPs** on HeLa cells was studied by standard MTT assay. The cytotoxicity of HeLa cells upon incubation with **TAT–TPDC NPs** and **TAT–PPDC NPs** in dark conditions was first evaluated. As shown in Fig. 6A, after 24 h incubation, no significant cytotoxicity is observed in the dark. However, after exposure to light irradiation, a dose-dependent cytotoxicity is observed in HeLa cells for both types of NPs. The half-maximal inhibitory concentrations (IC_50_) of **TAT–TPDC NPs** and **TAT–PPDC NPs** for HeLa cells are 3.44 and 1.28 μg mL^–1^, respectively. The lower IC_50_ of **TAT–PPDC NPs** relative to that for **TAT–TPDC NPs** should be attributed to the higher ^1^O_2_ quantum yield and more efficient light-induced ^1^O_2_ generation upon light irradiation for the former. The 2.6-fold lower IC_50_ of **TAT–TPDC NPs** is reckoned significant in cancer cell inhibition. Furthermore, to validate the exposure time and light power-dependent PDT, both **TAT–TPDC NP**- and **TAT–PPDC NP**-incubated HeLa cells were irradiated with light for different time durations or at different power densities. For example, when the cells were incubated with 1.0 μg mL^–1^ each of the NPs, followed by white light irradiation at a power density of 100 mW cm^–2^, the time required to reach 50% cell viability was 73 s for **TAT–PPDC NPs** and 113 s for **TAT–TPDC NPs**. By keeping the NP concentration the same at 1.0 μg mL^–1^, and the irradiation time as 60 s, varying the light power densities also results in different cell viabilities (Fig. 6C). Under all the tested conditions, **TAT–PPDC NPs** showed enhanced inhibition of cell viability as compared to **TAT–TPDC NPs**, which clearly demonstrates the importance of PS design in photodynamic therapy. These results also show that therapeutic efficiency can be regulated by controlling the laser irradiation time or the light power density.

Fluorescein isothiocyanate (FITC)-tagged Annexin V, a cell apoptosis indicator, was subsequently used to study whether cell apoptosis occurred in the PDT process. FITC-tagged Annexin V is commonly used to distinguish viable cells from apoptotic ones, as Annexin V can selectively bind to the exposed phosphatidylserines on the outer cytoplasmic membrane of apoptotic cells. As shown in Fig. S12,† after incubation of HeLa cells with **TAT–TPDC NPs** or **TAT–PPDC NPs** followed by light irradiation and FITC-tagged Annexin V staining, strong green fluorescence attributed to FITC is clearly observed on the cell membranes, indicating that the cells undergo the apoptosis process. On the other hand, no green fluorescence signal is observed in HeLa cells under dark conditions, indicating that **TAT–TPDC NPs** and **TAT–PPDC NPs** show low dark toxicity. The results indicate that the ^1^O_2_ clearly causes cell death.

## Conclusions

In this contribution, three AIEgen PSs of **TPDC**, **TPPDC** and **PPDC** were designed and synthesized with fine-tuned Δ*E*_ST_ *via* HOMO–LUMO engineering. Among the three AIEgens, **PPDC** shows the highest ^1^O_2_ quantum yield of 0.89, owing to its lowest Δ*E*_ST_ value (0.27 eV). This led to higher HeLa cell inhibition for **TAT–PPDC NPs** relative to **TAT–TPDC NPs** upon white light illumination. These observations validate our hypothesis that reducing the Δ*E*_ST_ values could yield higher ^1^O_2_ generation and more efficient PDT. Distinguished from traditional PSs that show ACQ characteristics with reduced ^1^O_2_ generation in aggregates (*e.g.* NPs), the AIEgen PSs developed in this work show strong fluorescence emission and efficient ^1^O_2_ generation in NPs, which make them highly favorable for image-guided cancer therapy. When the fine-tuning of the Δ*E*_ST_ approach is further extended to a large variety of fluorophores, such as BODIPY, rhodamine, and phthalocyanine, *etc.*, one would expect to develop new PSs with enhanced ^1^O_2_ generation for more efficient PDT.
