# Supplementary material for: Tuning the singlet-triplet energy gap: a unique approach to efficient photosensitizers with aggregation-induced emission (AIE) characteristics
Source: Chem Sci. 2015 Jul 17;6(10):5824–30. doi: 10.1039/c5sc01733e (PMC5520955; doi:10.1039/c5sc01733e)
Supplement: Supplementary file 1 [file SC-006-C5SC01733E-s001.pdf]

Electronic Supplementary Material (ESI) for Chemical Science.
This journal is © The Royal Society of Chemistry 2015

Supporting Information

Tuning the Singlet-Triplet Energy Gap: Unique Approach to Efficient Photosensitizers with Aggregation-Induced Emission (AIE) Characteristics

Shidang Xu,[‡†] *Youyong Yuan,*[‡ †] *Xiaolei Cai,*[†] *Chong-Jing Zhang,*[†] *Jing Liang,*[†] *Fang Hu,*[§] *Guanxin Zhang,*[§] *Deqing Zhang,*[§] *and Bin Liu*[*,†,#]

[†]Department of Chemical and Biomolecular Engineering, 4 Engineering Drive 4, National University of Singapore, Singapore, 117585

[#]Institute of Materials Research and Engineering, 3 Research Link, Singapore, 117602

[§]Beijing National Laboratory for Molecular Sciences, Organic Solids Laboratory, Institute of Chemistry, Chinese Academy of Sciences, Beijing 100190, China

Synthesis of DMTPBr. To the solution of 4-bromobenzophenone (5.3 g, 20.3 mmol) and 4,4'-dimethoxybenzophenone (3.8 g, 15.7 mmol) in dry THF (80 ml) was added zinc powder (5.9 g, 90.8 mmol). Then the suspension was cooled down to −78 ^{o}C. Titanium tetrachloride (5.0 ml) was added to the above mixture dropwise. After addition, the mixture was slowly warmed up to room temperature, followed by reflux for 8 hours. Then the mixture was cooled down in ice-water bath and saturated sodium bicarbonate aqueous solution (50 mL) was added slowly. The mixture was extracted with ethyl acetate (100 ml × 3). The combined organic phase was washed with brine (100 ml × 2), and dried over $MgSO_4$. Then the mixture was filtered and the filtrate was concentrated under reduced pressure. The desired residue was purified with chromatography (hexane/ethyl acetate = 100/1 to 30/1) to give the desired product (3.1 g, 41.8%). ^{1}H NMR (400 MHz, $CDCl_3$) δ 7.22 (d, J = 8.0 Hz, 2H), 7.10 (m, 3H), 7.00 (m, 2H), 6.87-6.94 (m, 6H), 6.67 (d, J = 8.8 Hz, 2H), 6.63 (d, J = 8.8 Hz, 2H), 3.76 (s, 3H), 3.73 (3, 3H); ^{13}C NMR (100 MHz, $CDCl_3$) δ 158.2, 158.0, 143.8, 143.1, 140.9, 137.8, 136.1, 135.9, 133.0, 132.5, 131.3, 130.8, 127.7, 126.2, 113.2, 113.0, 55.0.

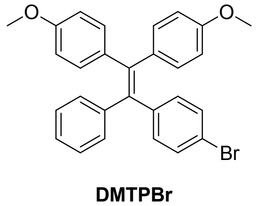

DMTPBr

Synthesis of PPAc. 4-acetylphenylboronic acid (1.04g, 6.36 mmol) and **DMTPBr** (2.00g, 4.24 mmol) were dissolved in THF (20.0 mL), and then 2 M aqueous K_2CO_3 solution (4.0 mL) and Aliquat 336 were added. The mixture was stirred for 40 min under an argon atmosphere at room temperature. Then the $Pd(PPh_3)_4$ catalyst was added and the reaction mixture was stirred at 75 ^{o}C for 16 h. After cooling to room temperature, the mixture was extracted with ethyl acetate (50mL×3). The combined organic phase was concentrated and purified by silica gel column chromatography

(hexane/ethyl acetate = 40/1-5/1). The product was dried under vacuum to afford compound **PPAc** as a yellow solid (1.80 g, 83% yield).^{1}H NMR (400 MHz, CDCl3) δ 7.92 (d, *J* = 8.3 Hz, 2H), 7.58 (d, *J* = 8.4 Hz, 2H), 7.32 (d, *J* = 8.3 Hz, 2H), 7.09 – 6.96 (m, 6H), 6.89 (dd, *J* = 15.0, 8.7 Hz, 4H), 6.58 (t, *J* = 8.6 Hz, 4H), 3.67 (s, 6H), 2.55 (s, 4H). ^{13}C NMR (100 MHz, $CDCl_3$) δ 197.6, 158.2, 158.1, 145.2, 144.5, 144.1, 140.7, 138.4, 137.0, 136.1, 135.6, 132.5, 131.9, 131.4, 128.9, 128.8, 127.7, 127.4, 126.8, 126.3, 126.2, 113.1, 113.0, 77.3, 77.0, 76.7, 55.0, 55.0, 26.6, 26.5. EI-MS, m/z: $[M+1]^+$ calcd 510.2, found 510.5.

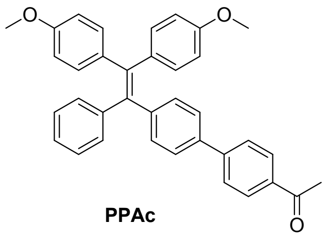

PPAc

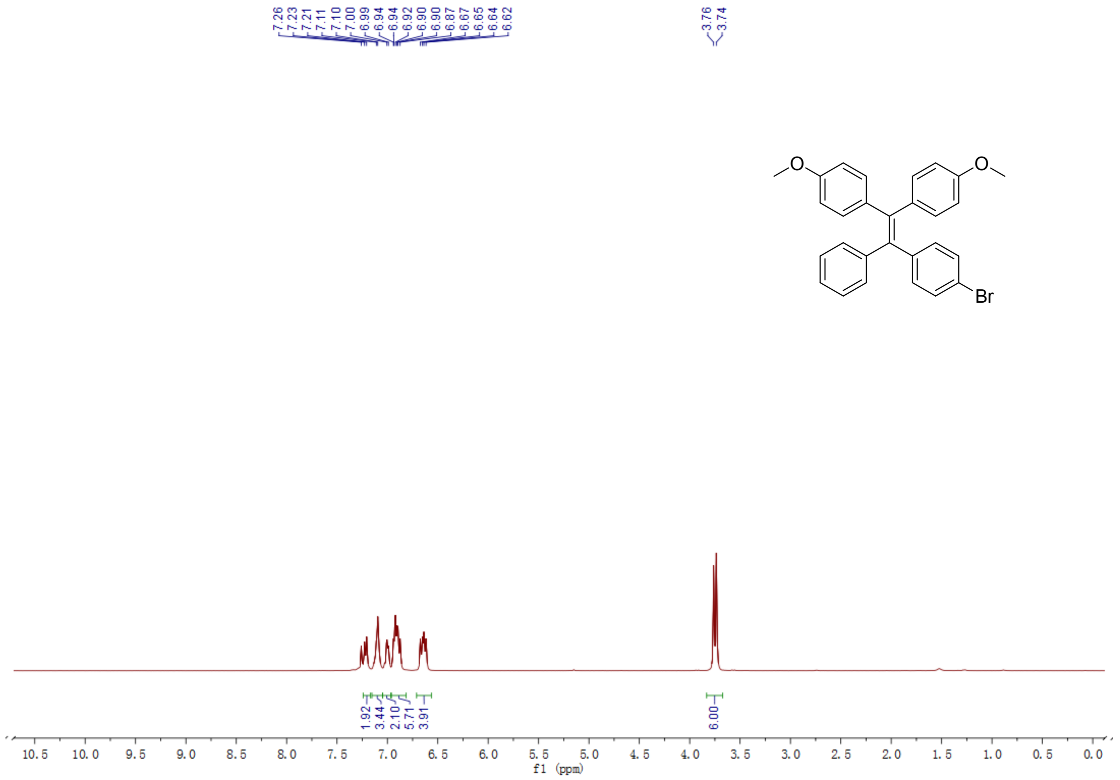

Figure S1. ^{1}H NMR spectrum of compound **DMTPBr** in $CDCl_3$.

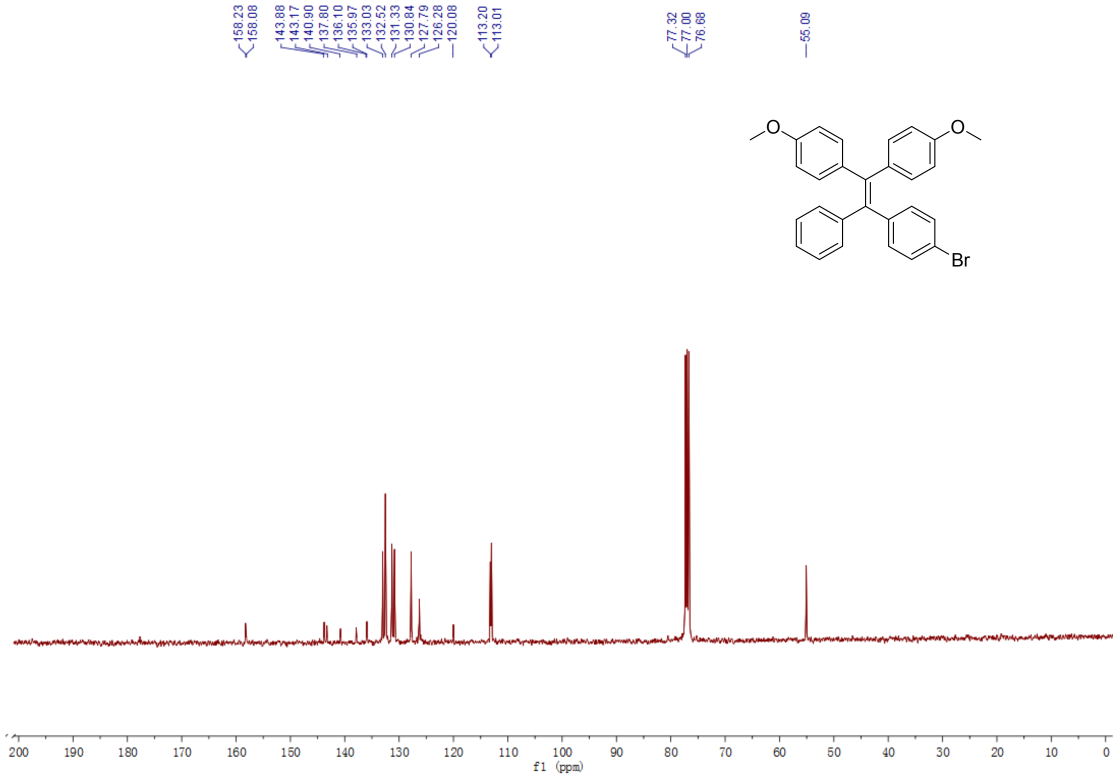

Figure S2. ^{13}C NMR spectrum of compound **DMTPBr** in $CDCl_3$.

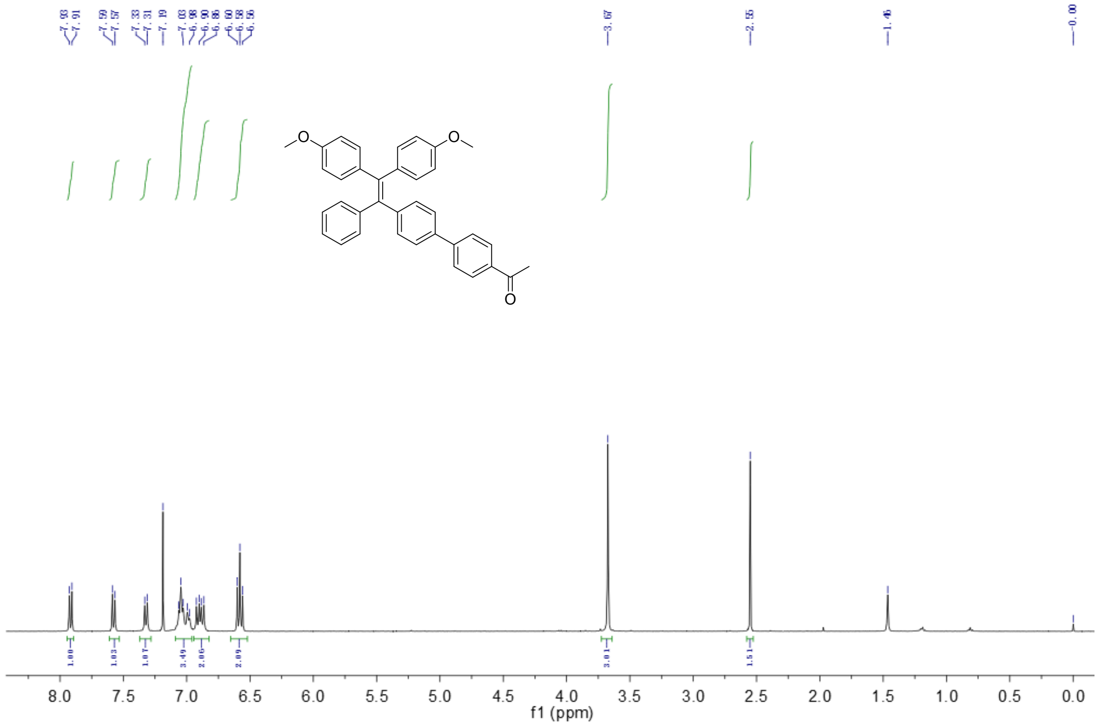

Figure S3. ^{1}H NMR spectrum of compound **PPAc** in $CDCl_3$.

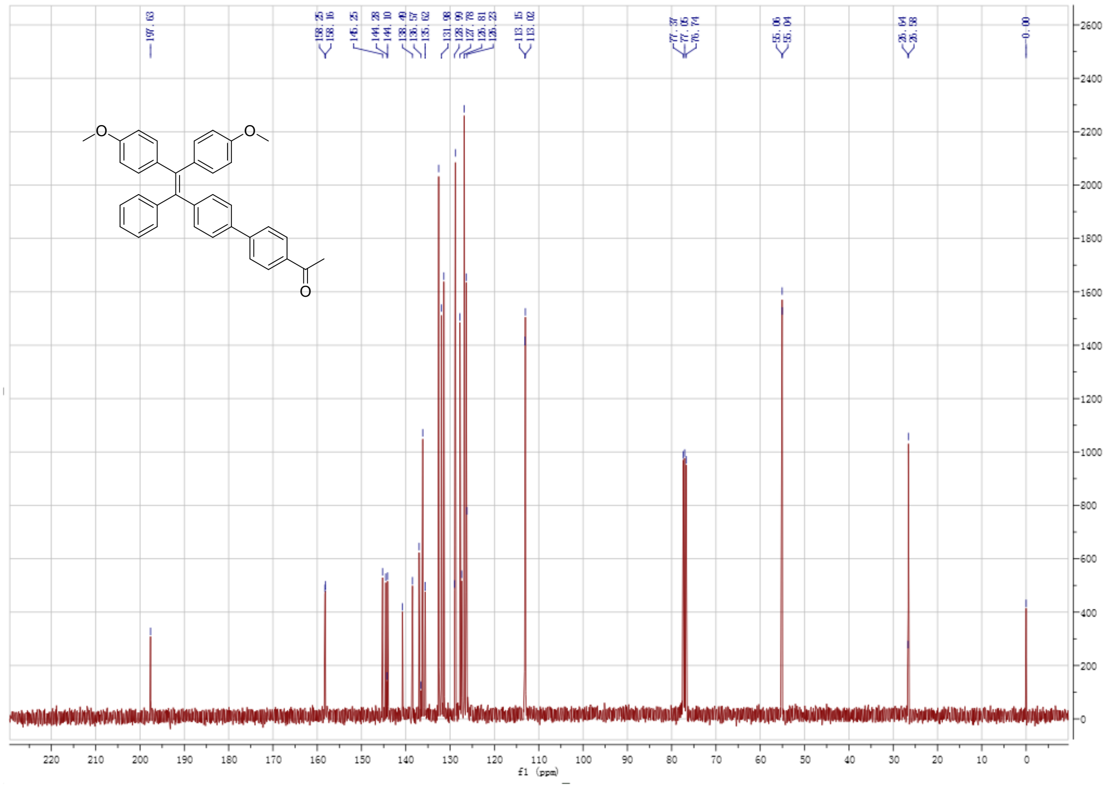

Figure S4. ^{13}C NMR spectrum of compound **PPAc** in $CDCl_3$.

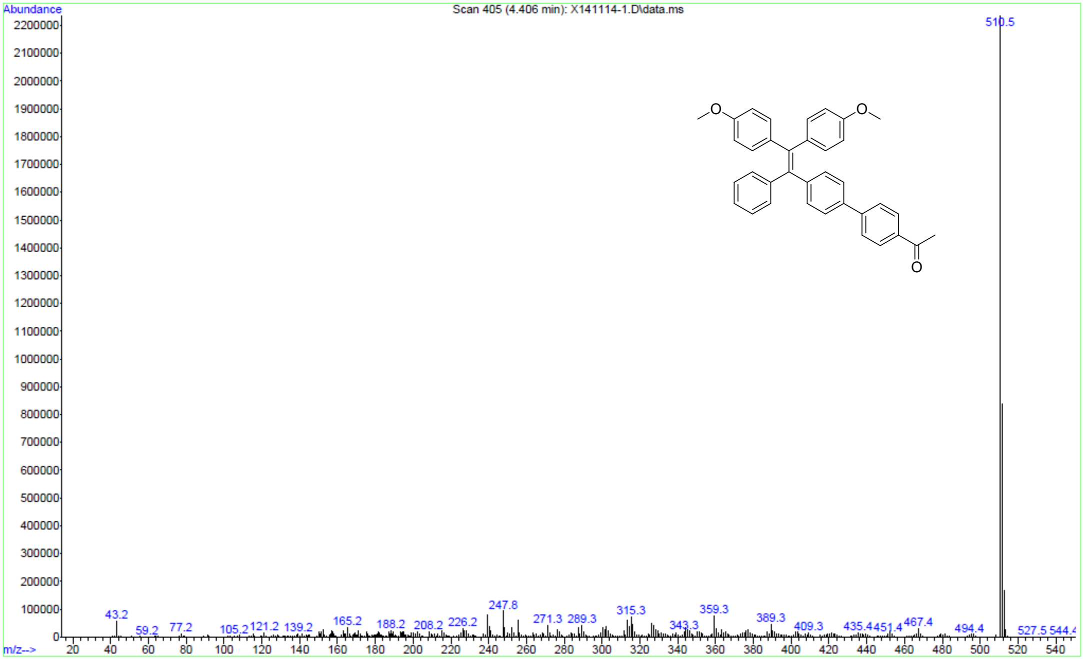

Figure S5. MS spectrum of compound **PPAc** in $CDCl_3$.

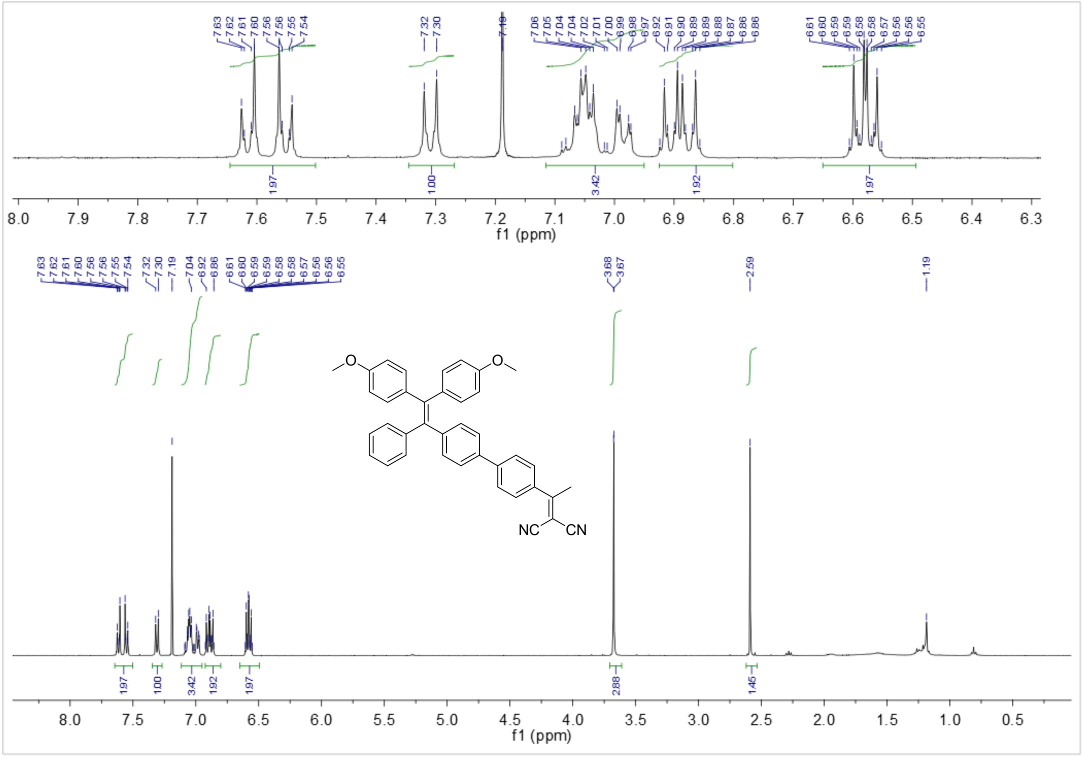

Figure S6 ^{1}H NMR spectrum of compound **PPDC** in $CDCl_3$.

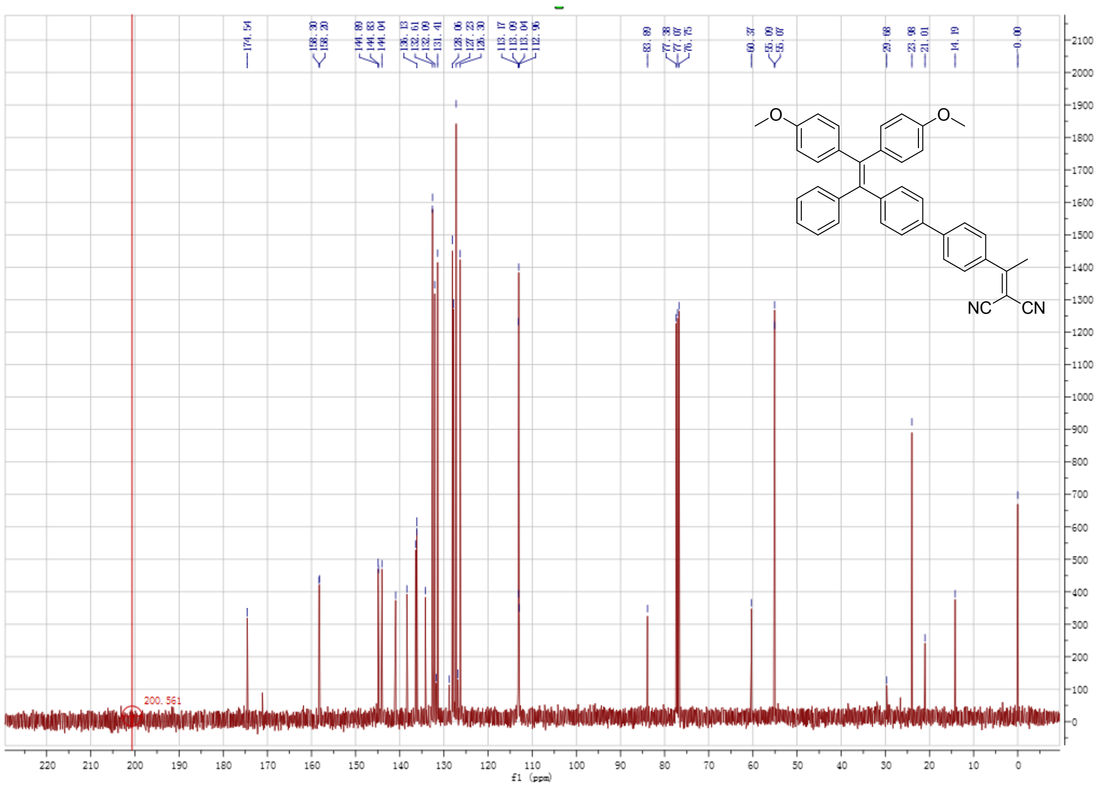

Figure S7. ^{13}C NMR spectrum of compound **PPDC** in $CDCl_3$.

```
File        :C:\MSDCHEM\1\DATA\OUTSIDE\CHBE\20141117\Snapshot\X141114-2.D
Operator    : SY
Acquired    : 17 Nov 2014  11:44     using AcqMethod 40_300.M
Instrument  :   Instrument #1
Sample Name: X141114-2
Misc Info   : ChBE A/P Liu Bin
Vial Number: 1
```

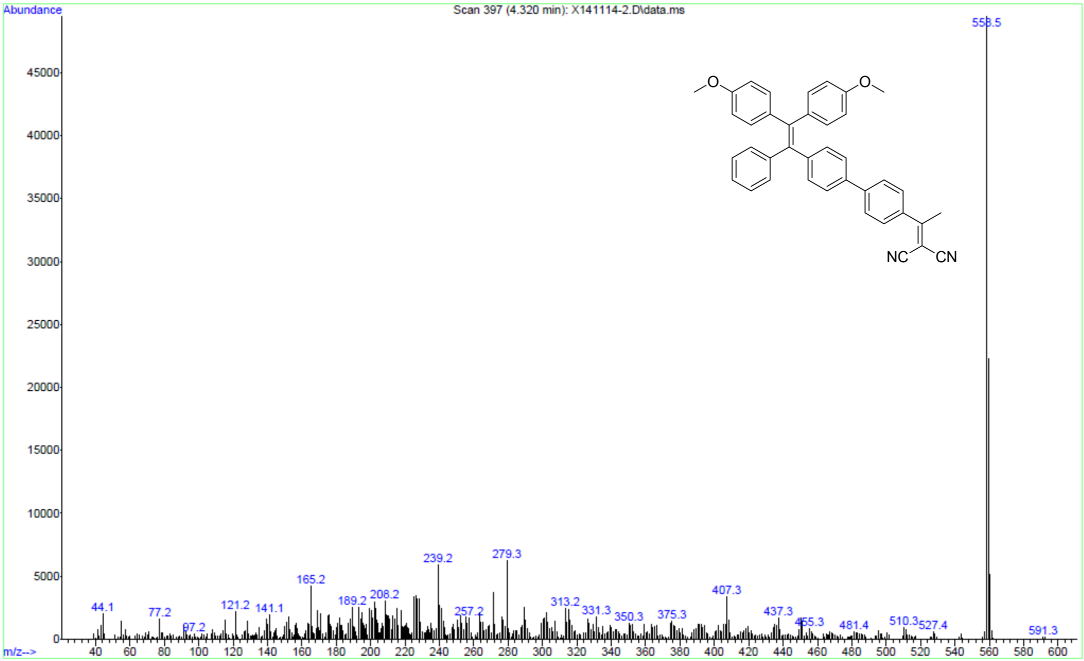

Figure S8. MS spectrum of compound **PPDC** in $CDCl_3$.

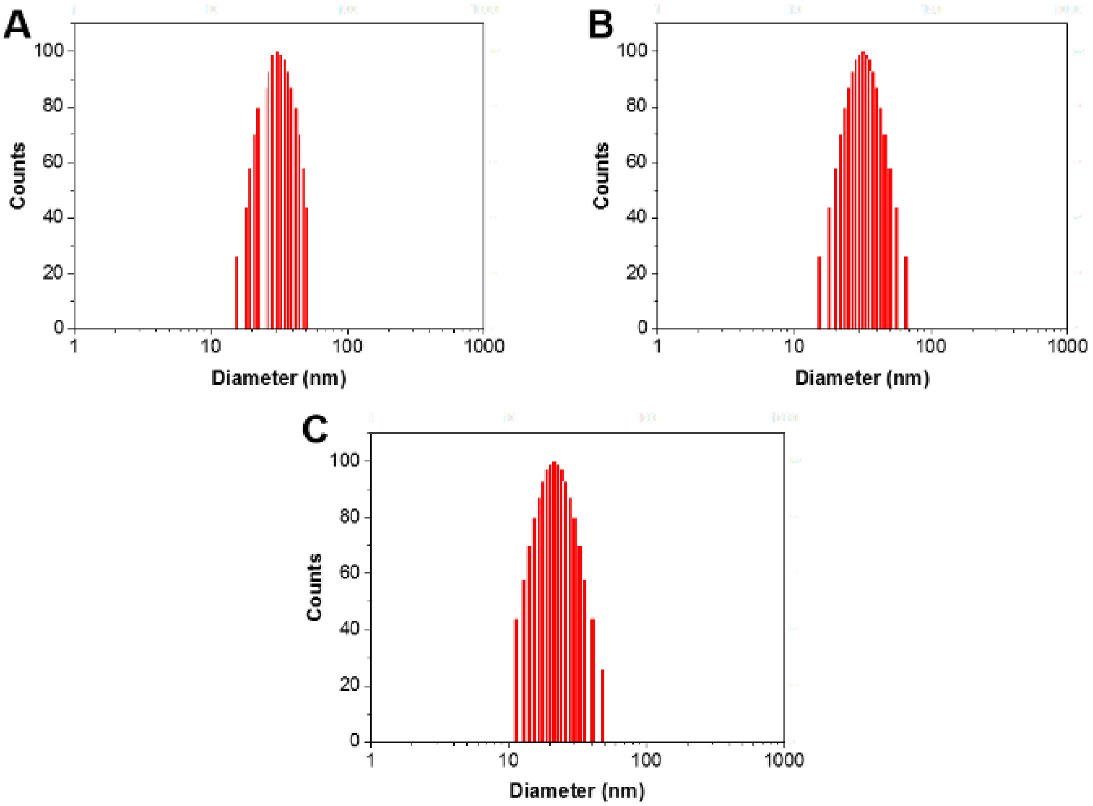

Figure S9. Size distributions of aggregates of 10 μM **TPDC** (A), **PPDC** (B) and **TPPDC** (C) in DMSO/water (v/v = 1/99).

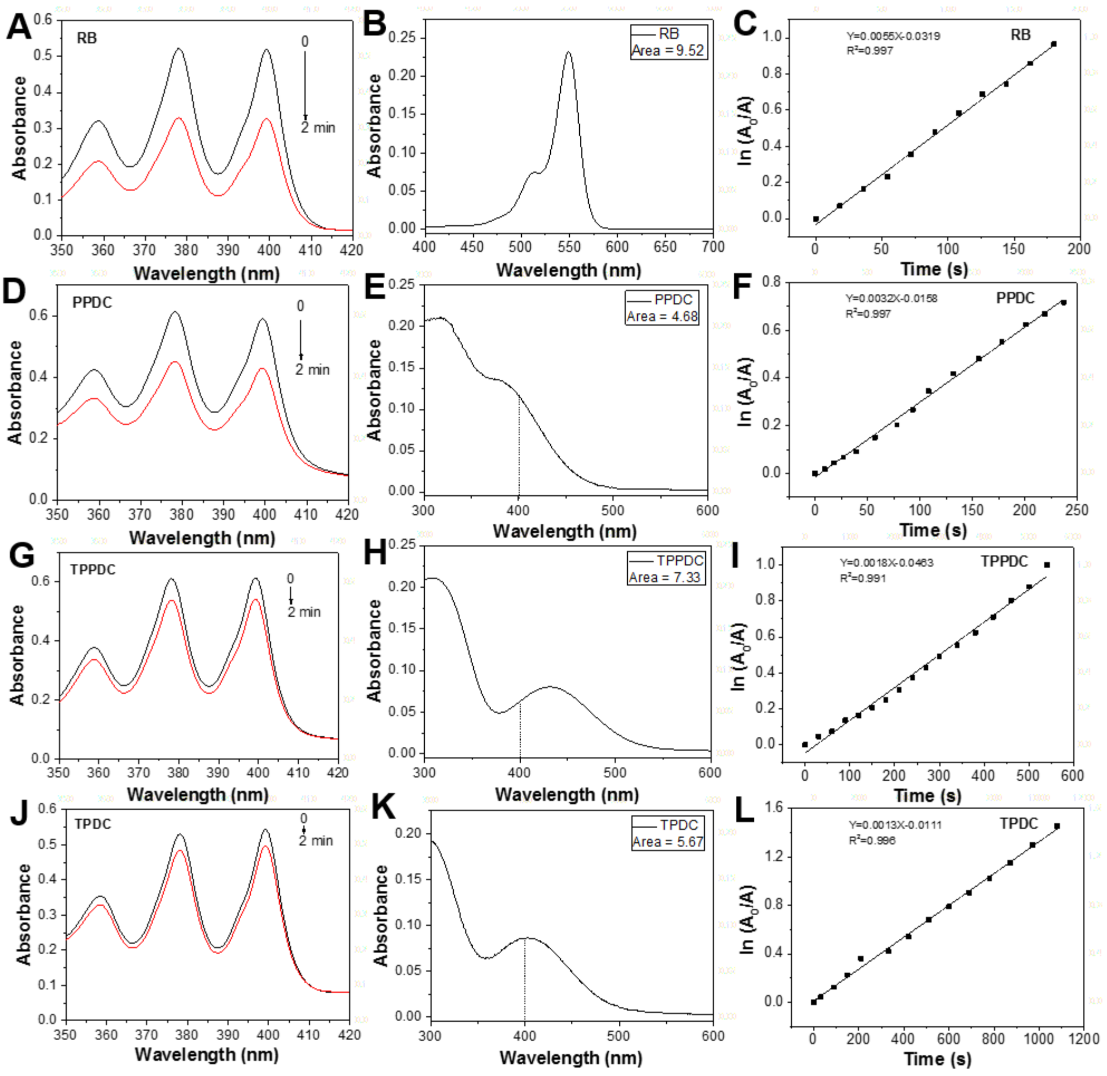

Figure S10. Chemical trapping measurements of the $^{1}O_2$ quantum yield. Photodegradation of ABDA with RB (A), **PPDC** (D), **TPPDC** (G) and **TPDC** (J); The absorption peak area of RB (B), **PPDC** (E), **TPPDC** (H) and **TPDC** (K); The decomposition rate constants of ABDA by RB (C), **PPDC** (F), **TPPDC** (I) and **TPDC** (L). To eliminate the inner-filter effect, the absorption maxima were adjusted to ~0.2 OD.

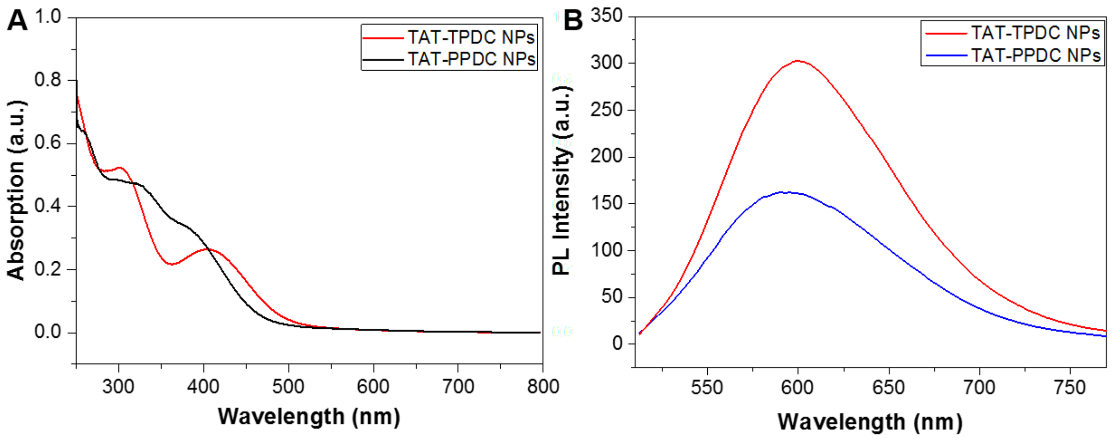

Figure S11. (A) UV-vis absorption and (B) PL spectra of **TAT-TPDC NPs** and **TAT-PPDC NPs**. E_x = 405 nm. Concentration: 10 μg/mL.

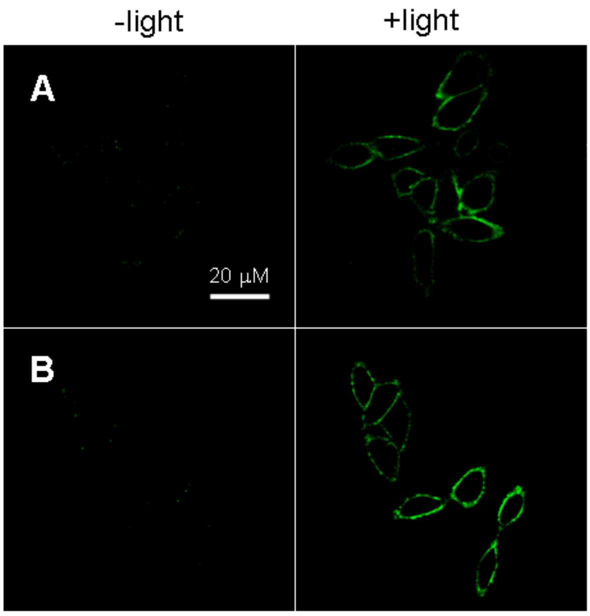

Figure S12. Cell apoptosis imaging using FITC-tagged Annexin V in HeLa cells incubated with **TAT-TPDC NPs** (A) and **TAT-PPDC NPs** (B) with or without light irradiation (0.25 W cm^{-2}). The green fluorescence is from FITC (E_x: 488 nm; E_m: 505−525 nm). All images share the same scale bar of 20 μm.

Geometry Data for **TPE** (S_0 optimization: unit Å)

C	-3.57082400	-3.06991100	0.40528900
C	-3.32920800	-2.01530200	1.28740300
C	-2.18296400	-1.23403800	1.15159800
C	-1.25157300	-1.49067700	0.13133500
C	-1.49745700	-2.57019100	-0.73526700
C	-2.64951600	-3.34387800	-0.60742300
C	-0.00000500	-0.68306800	-0.00000200
C	-0.00000300	0.68306600	0.00000000
C	1.25156600	-1.49067500	-0.13133700
C	1.25156900	1.49067000	0.13133600
C	-1.25157000	1.49067900	-0.13133600
C	2.18295100	-1.23404200	-1.15160700
C	3.32919700	-2.01530300	-1.28741100
C	3.57082200	-3.06990300	-0.40528900
C	2.64952000	-3.34386400	0.60743100
C	1.49745800	-2.57018000	0.73527400
C	-2.18296800	1.23403700	-1.15159100
C	-3.32920900	2.01530600	-1.28739500
C	-3.57081500	3.06992300	-0.40528800
C	-2.64949900	3.34389300	0.60741600
C	-1.49744300	2.57020200	0.73525900
C	1.49747000	2.57016700	-0.73528200
C	2.64953300	3.34384800	-0.60743900
C	3.57082700	3.06989300	0.40529000
C	3.32919300	2.01530200	1.28742000
C	2.18294500	1.23404300	1.15161500
H	-4.46508200	-3.67730300	0.51046900
H	-4.03294400	-1.80153600	2.08685500
H	-2.00104700	-0.41597800	1.84060900
H	-0.77849500	-2.79718800	-1.51665500
H	-2.82541200	-4.16506900	-1.29654900
H	2.00102700	-0.41598900	-1.84062500
H	4.03292800	-1.80154200	-2.08686900
H	4.46508100	-3.67729300	-0.51046800
H	2.82542300	-4.16504800	1.29656400
H	0.77850100	-2.79717200	1.51666800
H	-2.00106000	0.41597100	-1.84059800
H	-4.03295000	1.80153800	-2.08684200
H	-4.46507000	3.67731900	-0.51046700
H	-2.82538700	4.16509000	1.29653800
H	-0.77847400	2.79720100	1.51664100
H	0.77851800	2.79715400	-1.51668300
H	2.82544300	4.16502500	-1.29657800
H	4.46508700	3.67728200	0.51046900
H	4.03291700	1.80154700	2.08688500
H	2.00101400	0.41599600	1.84063800

Geometry Data for **TPS** (S_0 optimization: unit Å)

C	-0.75595200	0.27721900	0.02428300
C	0.75595000	0.27721800	-0.02428500
C	1.35772800	-0.94876200	-0.07842900
Si	-0.00000200	-2.25599300	0.00000600
C	-1.35773100	-0.94876000	0.07842700

C	-1.50823400	1.56619100	0.01604000
C	1.50823600	1.56618800	-0.01604100
C	2.80664500	-1.23634500	-0.01472400
C	-2.80664800	-1.23634200	0.01471800
C	1.35579400	2.49640300	1.02523900
C	2.08824300	3.68264900	1.03475500
C	2.97650800	3.96940900	-0.00332900
C	3.13014300	3.05809700	-1.04922100
C	2.40580800	1.86689300	-1.05304600
C	-2.40580700	1.86689600	1.05304500
C	-3.13013800	3.05810200	1.04922200
C	-2.97650000	3.96941600	0.00333100
C	-2.08823500	3.68265500	-1.03475300
C	-1.35578800	2.49640700	-1.02523800
C	-3.36335800	-2.22188400	0.85258500
C	-4.71761700	-2.54701000	0.78779000
C	-5.54911700	-1.90763300	-0.13227200
C	-5.01162200	-0.93947700	-0.98351400
C	-3.66106400	-0.60651300	-0.91136900
C	3.66106100	-0.60652700	0.91137000
C	5.01161900	-0.93949200	0.98351200
C	5.54911400	-1.90763700	0.13225700
C	4.71761400	-2.54700200	-0.78781300
C	3.36335500	-2.22187600	-0.85260400
C	-0.19333900	-3.34329900	-1.53588300
C	0.19333600	-3.34326500	1.53592000
H	0.66046300	2.28683900	1.83196000
H	1.96097400	4.38590200	1.85283700
H	3.54215400	4.89662500	0.00189900
H	3.81651500	3.27293100	-1.86334600
H	2.53337000	1.15625900	-1.86359600
H	-2.53337100	1.15626100	1.86359400
H	-3.81651000	3.27293700	1.86334700
H	-3.54214200	4.89663300	-0.00189500
H	-1.96096300	4.38591000	-1.85283300
H	-0.66045800	2.28684300	-1.83195900
H	-2.72660400	-2.72218300	1.57660600
H	-5.12193400	-3.30306300	1.45514200
H	-6.60299700	-2.16382100	-0.18959600
H	-5.64729800	-0.44207400	-1.71081200
H	-3.25510500	0.14278900	-1.58204700
H	3.25510100	0.14276800	1.58205700
H	5.64729500	-0.44209800	1.71081600
H	6.60299400	-2.16382600	0.18957700
H	5.12193100	-3.30304700	-1.45517500
H	2.72660000	-2.72216500	-1.57663100
H	-0.18931600	-2.74463900	-2.45163200
H	-1.14622200	-3.88244300	-1.49315900
H	0.60689800	-4.08698100	-1.60764700
H	-0.60690000	-4.08694600	1.60770100
H	0.18930900	-2.74458200	2.45165400
H	1.14622000	-3.88240900	1.49321100

Geometry Data for **TPDC** (S_0 optimization: unit Å)

C	5.64169400	-0.15306100	0.85960800

C	4.70824900	-0.98307600	1.50178000
C	3.35856300	-0.88500500	1.19666700
C	2.88599500	0.04306300	0.24714400
C	3.83258400	0.87830700	-0.36611900
C	5.19552200	0.78348600	-0.08189900
C	1.43278600	0.18136700	-0.05448800
C	0.92496000	1.58282100	-0.09836700
C	0.62341300	-0.89818200	-0.28944400
C	-0.86365100	-0.80006200	-0.30660700
C	1.16071600	-2.26763900	-0.56001900
C	0.15624800	2.05004700	-1.17443500
C	-0.29923200	3.36794200	-1.23759800
C	0.01206300	4.25737800	-0.20129900
C	0.79136400	3.81264900	0.87974100
C	1.24769100	2.50329900	0.91935000
C	0.67005300	-3.38364600	0.14098400
C	1.14958700	-4.66572100	-0.12679700
C	2.11633800	-4.86186200	-1.11610300
C	2.59847500	-3.76500400	-1.83457100
C	2.12581300	-2.48200300	-1.55935800
C	-1.61492300	-1.42109800	-1.32275200
C	-3.00133900	-1.35297300	-1.33929600
C	-3.70738200	-0.70744100	-0.30330600
C	-2.95772300	-0.09769200	0.72329300
C	-1.57022900	-0.13973600	0.71598400
O	6.94292600	-0.32727100	1.22480600
C	7.93265200	0.50073800	0.61610400
O	-0.37995700	5.56149100	-0.15323000
C	-1.16943100	6.07088700	-1.22692200
C	-5.17570300	-0.64388800	-0.33825100
C	-5.95100300	-0.71414200	0.79790600
C	-5.83557500	-0.50938500	-1.68314600
C	-5.44839200	-0.99881400	2.10899200
N	-5.10600600	-1.25137500	3.19283900
C	-7.37634800	-0.57387500	0.74935300
N	-8.53544700	-0.46196300	0.74063600
H	5.06354100	-1.69142800	2.24340800
H	2.65207600	-1.52897600	1.70936800
H	3.50124200	1.61493000	-1.09154900
H	5.89082000	1.43988900	-0.59086400
H	-0.08469700	1.37352900	-1.98796400
H	-0.88132600	3.68799700	-2.09314300
H	1.02939800	4.51308300	1.67386900
H	1.85583200	2.17879200	1.75804900
H	-0.08543000	-3.24134000	0.90818800
H	0.76636800	-5.51196500	0.43602800
H	2.48567800	-5.86053800	-1.32939200
H	3.34008100	-3.90857300	-2.61514600
H	2.50124300	-1.63449100	-2.12378300
H	-1.10025500	-1.95104400	-2.11740600
H	-3.53943800	-1.83243900	-2.14928000
H	-3.46049800	0.44410400	1.51566500
H	-1.02012100	0.34740700	1.51320500
H	7.75509100	1.56044200	0.83170000
H	8.88514600	0.19808200	1.05161200
H	7.96672300	0.35295900	-0.46924800
H	-0.63166200	6.01618700	-2.18025900

H	-1.36743800	7.11497100	-0.98362900
H	-2.11978900	5.53245200	-1.31592200
H	-6.90098300	-0.29441400	-1.60329800
H	-5.35145700	0.27729600	-2.26760100
H	-5.72673800	-1.44336500	-2.24630700

Geometry Data for **TPPDC** (S_0 optimization: unit Å)

C	-6.41482536	0.01044020	0.71196803
C	-5.52467721	-0.88831170	1.31212790
C	-4.15955559	-0.79119053	1.04316515
C	-3.64271046	0.18987345	0.18528769
C	-4.55612841	1.09805950	-0.38755259
C	-5.91764222	1.00689476	-0.14175208
C	-2.18020712	0.31275032	-0.07323273
C	-1.63817295	1.69972560	-0.00051509
C	-1.39074605	-0.76699125	-0.37098585
C	0.09847645	-0.69509109	-0.35106605
C	-1.94783461	-2.10034668	-0.75495923
C	-1.98526498	2.55833620	1.06251806
C	-1.49344703	3.85247539	1.13586439
C	-0.65087583	4.34567854	0.12700689
C	-0.31487892	3.52111512	-0.95328301
C	-0.80774591	2.21720658	-1.00487112
C	-2.92115550	-2.21918709	-1.76175637
C	-3.40801562	-3.46808134	-2.14280974
C	-2.93283367	-4.62740677	-1.52664725
C	-1.95905280	-4.52691189	-0.53144150
C	-1.46574295	-3.27800330	-0.15704137
C	0.79251781	-0.13951879	0.74055065
C	2.17929276	-0.10948855	0.77050017
C	2.93786744	-0.60507831	-0.30922230
C	2.24814198	-1.18173526	-1.39215529
C	0.86055820	-1.24296918	-1.39956765
O	-7.76572418	0.00745646	0.89704094
C	-8.32582707	-0.98034935	1.74897276
O	-0.22499496	5.63035406	0.28636972
C	0.62708591	6.18517895	-0.70573056
C	4.41087379	-0.52369587	-0.27228696
C	5.14167631	-0.17818339	-1.39030453
C	5.08461773	-0.82086332	1.01476074
C	4.66779984	-1.92591151	1.78043347
C	5.29520987	-2.22940768	2.98537440
C	6.33046842	-1.42087083	3.45975002
C	6.73804572	-0.30949991	2.72036448
C	6.12541663	-0.01286880	1.50523061
C	6.57292934	-0.25116119	-1.44655956
N	7.73134025	-0.30442233	-1.54989337
C	4.54306979	0.29883020	-2.60320576
N	4.10914547	0.71191883	-3.60147528
H	-5.87770654	-1.65761077	1.98837127
H	-3.48086923	-1.49294752	1.51620922
H	-4.18641559	1.88096708	-1.04228395
H	-6.61971882	1.69873373	-0.59524508
H	-2.64418085	2.19523828	1.84524862
H	-1.74969519	4.50707210	1.96237609

H	0.31829751	3.87922935	-1.75603675
H	-0.54348614	1.59133996	-1.85075174
H	-3.29340554	-1.32230694	-2.24579848
H	-4.15614784	-3.53581738	-2.92745206
H	-3.31268658	-5.60036316	-1.82424629
H	-1.57976066	-5.42263469	-0.04770134
H	-0.70169605	-3.20892303	0.61193798
H	0.23014963	0.27038543	1.57214858
H	2.68725371	0.32016019	1.62745722
H	2.80214605	-1.60741861	-2.22122221
H	0.35313829	-1.71451222	-2.23473965
H	-7.94516554	-0.89354026	2.77454248
H	-8.12817371	-1.99399761	1.37802547
H	-9.40190494	-0.80171156	1.74973081
H	0.84449628	7.20304017	-0.37951176
H	0.13739050	6.21745310	-1.68700959
H	1.56630916	5.62478680	-0.79204240
H	3.86171581	-2.55326537	1.41482791
H	4.97429058	-3.09569644	3.55575349
H	6.81369626	-1.65338081	4.40397394
H	7.53339997	0.33030067	3.08966151
H	6.44083617	0.85981210	0.94449924

Geometry Data for **PPDC** (S_0 optimization: unit Å)

C	7.23864300	-0.43716200	-0.61107000
C	6.28188200	-1.14129500	-1.35157000
C	4.92501800	-0.94469000	-1.09168000
C	4.48196300	-0.05352800	-0.10428000
C	5.46391100	0.66175100	0.61084600
C	6.81685700	0.46876700	0.37414500
C	3.03008700	0.17774200	0.14844200
C	2.62889500	1.61144700	0.24821800
C	2.13378200	-0.84878100	0.28384000
C	0.65641500	-0.63962800	0.24908600
C	2.55624800	-2.27003600	0.48069100
C	3.09599300	2.55665200	-0.67707500
C	2.74919300	3.90600700	-0.59245700
C	1.93136900	4.34546700	0.45538400
C	1.47329000	3.42045100	1.40593300
C	1.81679100	2.08100800	1.29960700
C	3.49216100	-2.62219800	1.46829800
C	3.85292100	-3.95320500	1.67182900
C	3.28599300	-4.96375300	0.89238600
C	2.34716700	-4.63140700	-0.08629600
C	1.97961900	-3.30092100	-0.28241600
C	0.04364300	0.13644000	-0.75083500
C	-1.33679100	0.29149200	-0.79942000
C	-2.17303400	-0.32679100	0.14704800
C	-1.56348800	-1.11335800	1.14026500
C	-0.18294200	-1.27250600	1.18401200
O	8.58834300	-0.55160400	-0.77366600
C	9.07219200	-1.45918400	-1.75107700
O	1.53473300	5.63718900	0.64486400
C	1.98473600	6.62040600	-0.27371300
C	-3.64268000	-0.16303700	0.09189300
C	-4.22616600	1.04762400	-0.32490600

C	-5.60452700	1.20517200	-0.37018800
C	-6.47254500	0.14843200	-0.02722400
C	-5.88908000	-1.06002000	0.40106500
C	-4.50946700	-1.20711800	0.46092100
C	-7.93139300	0.34627600	-0.07688100
C	-8.81599700	-0.64741300	-0.42082300
C	-8.45565400	1.72226400	0.25250000
C	-10.23389200	-0.42607000	-0.40329900
N	-11.38550700	-0.25699600	-0.39110800
C	-8.43803100	-1.95389400	-0.87606100
N	-8.18575200	-3.02258700	-1.26164300
H	6.57664700	-1.83616500	-2.12977400
H	4.19473100	-1.49733600	-1.67426100
H	5.15494800	1.37406000	1.37052000
H	7.57031500	1.01094600	0.93720200
H	3.74103900	2.23101600	-1.48814600
H	3.12386000	4.59800200	-1.33808100
H	0.85551300	3.77956600	2.22327500
H	1.45829700	1.38009700	2.04684300
H	3.93625200	-1.84199600	2.07869700
H	4.57519400	-4.20153500	2.44526300
H	3.56778300	-6.00128400	1.05115500
H	1.89612200	-5.40979600	-0.69644100
H	1.24032200	-3.05234000	-1.03892600
H	0.66154500	0.61293200	-1.50494900
H	-1.77611600	0.87021300	-1.60722200
H	-2.17558300	-1.58572300	1.90357400
H	0.25848800	-1.88833200	1.96214600
H	10.16097600	-1.39832400	-1.70320600
H	8.75621200	-2.48871100	-1.53714900
H	8.73797100	-1.18270600	-2.75989600
H	1.55854600	7.56574700	0.06710700
H	3.08003600	6.69743900	-0.27861900
H	1.63431000	6.41188700	-1.29331300
H	-3.58826500	1.88579800	-0.58854400
H	-6.01036900	2.15875300	-0.69202200
H	-6.51644900	-1.88810000	0.70894700
H	-4.09608100	-2.15900400	0.77949400
H	-8.24174100	2.41760200	-0.56959900
H	-9.53566200	1.72398800	0.40832900
H	-7.96312200	2.11728700	1.14644200
